# Atropia Used Hypodermically

**Published:** 1869-04

**Authors:** 

**Affiliations:** Pennsylvania Hospital Reports


Atropia used Hypodermically.—Dr. Da Costa (*Pennsylvania Hospital Reports*, art. xxi), reports two instances of the successful use of the hypodermic injection of sulph. of atropia, in cases of muscular rheumatism. The first, a servant girl, had been suffering for some six weeks previous to admission, with pain in the various joints, and swelling of the left knee and ankles; Most of the time since, has been confined to bed. At present, there is no swelling of any joint, but stiffness, and pain on motion in left hip, knee, and ankle. She was treated with iodide potassa and pulv. Doveri. Soon after admission, a stiff neck was noticed, which in a few days presented the ungainly appearance of marked torticollis. Frictions and ordinary remedies were of no avail. An injection of $\frac{1}{50}$ of a grain of sulph. of atropia, was now used just over the rigid parts resulting at once in a decided amelioration. The injections were repeated three successive days, and were followed by perfect relief. They were afterwards used over the stiff and painful joints with no beneficial result. A complete recovery took place in this case by persisting with the constitutional treatment.

The second case was one of lumbago, in a sailor, æt 40 years. Treatment with diaphoretics, opiates, and friction, was without effect. The muscles in the lumbar region, along the spine, were quite rigid and painful on pressure. Somewhat less than $\frac{1}{40}$ of a grain of atropia was injected near the affected part. The costitutional effect was marked. In about fifteen minutes the man became giddy; his throat very dry; his pupils dilated. This state lasted some time and alarmed him; but when it had passed off, the pain and rigidity of the muscles were gone; nor did they return although soon after he went upon a voyage in which he was exposed to bad weather.

